# *Aploneura lentisci* (Homoptera: Aphididae) and Its Interactions with Fungal Endophytes in Perennial Ryegrass (*Lolium perenne*)

**DOI:** 10.3389/fpls.2016.01395

**Published:** 2016-09-16

**Authors:** Alison J. Popay, Neil R. Cox

**Affiliations:** Ruakura Research Centre, AgResearchHamilton, New Zealand

**Keywords:** *Epichloë festucae* var. *lolii*, root aphid, populations, plant growth, endophyte strain

## Abstract

*Aploneura lentisci* Pass. is endemic to the Mediterranean region where it is holocyclic, forming galls on its primary host, *Pistacia lentiscus* and alternating over a 2-year period between *Pistacia* and secondary hosts, principally species of Gramineae. This aphid is widely distributed in Australia and New Zealand on the roots of the common forage grasses, ryegrass (*Lolium* spp.) and tall fescue (*Schedonorus phoenix*) where it exists as permanent, anholocyclic, parthenogenetic populations. Previous studies have indicated that infestations of *A. lentisci* significantly reduce plant growth and may account for differences in field performance of *Lolium perenne* infected with different strains of the fungal endophyte *Epichloë festucae* var. *lolii*. These obligate biotrophs protect their host grasses from herbivory via the production of alkaloids. To confirm the hypothesis that growth of *L. perenne* is associated with the effect of different endophyte strains on aphid populations, herbage and root growth were measured over time in two pot trials that compared three fungal endophyte strains with an endophyte-free control. In both pot trials, aphid numbers were lowest on plants infected with endophyte strain AR37 at all sampling times. In plants infected with a common toxic strain naturalized in New Zealand, aphid numbers overall were lower than on uninfected plants or those infected with strain AR1, but numbers did not always differ significantly from these treatments. Populations on AR1-infected plants were occasionally significantly higher than those on endophyte-free. Cumulative foliar growth was reduced in AR1 and Nil treatments relative to AR37 in association with population differences of *A. lentisci* in both trials and root dry weight was reduced in one trial. In four Petri dish experiments survival of *A. lentisci* on plants infected with AR37 declined to low levels after an initial phase of up to 19 days during which time aphids fed and populations were similar to those on plants without endophyte. Aphids on AR37-infected plants became uncoordinated in their movement and developed tremors before dying suggesting a neurotoxin was responsible for their mortality. Results support the hypothesis that differences in *A. lentisci* populations due to endophyte infection status and strain affects plant growth.

## Introduction

There is an abundance of literature on the interactions between above-ground herbivores and their host plants, but comparatively little on root herbivores (Brown and Gange, 1990; Hunter, 2001), despite the profound effects that the latter can have on plant growth and physiology, and on the determination and regulation of soil communities (Anderson, 1987; Brown and Gange, 1990; Hunter, 2001; Wardle, 2002). The consequences that root herbivory have for individual plants, depending on the type of feeding and its severity, include reductions in above and below-ground plant growth, changes in biomass allocation, and effects on nutrient acquisition, water relations and physiological and morphological parameters of the plant (Brown and Gange, 1990; Hunter, 2001; Wardle, 2002). At the community level, root herbivory may alter plant competitiveness and diversity and the rate and direction of plant succession (Brown and Gange, 1990; Hunter, 2001; Wardle, 2002). As a major component of the soil foodweb, root herbivory also has major repercussions for soil microbial and invertebrate populations (Bardgett et al., 1999; Denton et al., 1999; Wardle, 2002).

Species of *Lolium* and *Festuca* are often infected with asexual clavicipitaceous endophytic fungi belonging to the genus *Epichloë* [previously *Neotyphodium* (Leuchtmann et al., 2014)]. These endophytes are obligate biotrophs and form, in most cases, a mutualistic relationship with their hosts in which they produce secondary metabolites that are deterrent or toxic to herbivorous insects (Popay and Bonos, 2005). There is no external stage and they are transmitted via seed. Much of the research into the effects of the *Epichloë* infection on insect herbivores has focused on those that feed above-ground. In part this relates to the location of endophyte infection in the meristematic and basal leaf sheath tissue along with the alkaloids that are also concentrated in above-ground tissues (Ball et al., 1997a,b; Lane et al., 1997) but also reflects the difficulties inherent in monitoring below-ground herbivory.

The particular alkaloids produced by *Epichloë* fungi are a characteristic of each different strain (Lane et al., 2000), although several factors moderate the quantities that are produced. These factors include plant genotype (Ball et al., 1995a,b; Easton et al., 2002), nutrient status (Rottinghaus et al., 1991; Azevedo et al., 1993) and environmental and seasonal factors (Ball et al., 1995a,b; Hennessy et al., 2016). Location of alkaloids within plants, however, appears to be mainly an attribute of the compounds themselves (Ball et al., 1995a, 1997a,b; Keogh et al., 1996; Lane et al., 1997) although this may also be modified to a degree by plant genotype (Popay et al., 2003).

In New Zealand pastures, there is a high incidence of endophyte infection of ryegrass by naturalized strains of the fungus (*Epichloë* festucae var. *lolii*; referred to here as Common Toxic (CT) but also known as wild-type or standard endophyte) that share a common chemical profile (Easton, 1999). Of the alkaloids they produce, ergovaline and lolitrem B are toxic to grazing mammals (Fletcher and Easton, 1997) as well as having effects on insect herbivores (Popay and Bonos, 2005); a third alkaloid, peramine, is a powerful deterrent to a major pest *Listronotus bonariensis* (Rowan et al., 1990) with no known effect on mammals (Fletcher, 1999; Tapper and Latch, 1999). In order to resolve the animal health problems associated with infection of ryegrass by the CT strains while retaining the anti-insect properties that infection provides, endophytes with different metabolic profiles have been investigated (Tapper and Latch, 1999). One of these, AR1, which produces peramine but not the mammalian toxins lolitrem B and ergovaline, was made commercially available to New Zealand farmers in 2001. In 2007, a second endophyte strain, AR37, which lacks the ability to produce peramine, ergovaline or lolitrem B was also commercially released to farmers. This strain produces indole-diterpenoid compounds, related to lolitrem B, known as epoxy-janthitrems (Finch et al., 2012). The role these particular compounds have for animal health and on insect pests is still being defined.

In New Zealand, fungal endophyte infection is necessary for plant survival in some areas and can considerably improve *Lolium perenne* growth, effects which have been attributed to protection against insect herbivory that the endophyte confers (Popay et al., 1999; Hume et al., 2007, 2009; Popay and Hume, 2011; Thom et al., 2014). In Australia, infection of *L. perenne* has delivered similar benefits for plant performance although the role of insect pests has been less well studied (Lowe et al., 2008; Hume and Sewell, 2014). The CT strain reduces predation of ryegrass by three pests, Argentine stem (*Listronotus bonariensis*), African black beetle (*Heteronychus arator*) and pasture mealybug (*Balanococcus poae*), of which Argentine stem weevil is the most significant. AR1 provides a similar spectrum of effects except it has a much weaker effect on African black beetle whereas AR37, in addition to these pests, also reduces populations of porina caterpillars (*Wiseana* spp.) and a root aphid, *Aploneura lentisci*. In trials comparing the agronomic benefits of these different strains in the same cultivar, AR37 has consistently out-performed both the CT and AR1 strain with those advantages attributed to the strong effects AR37 has in suppressing populations of *A. lentisci* (Hume et al., 2007; Thom et al., 2014). Perennial ryegrass infected with the endophyte strain AR37 also significantly reduced infestations of a root aphid *A. lentisci* in a pot trial with associated increases in plant growth (Popay and Gerard, 2007). This aphid is also adversely affected by *Epichloë* infection of tall fescue (*Schedonorus phoenix*) and meadow fescue (*L. pratense*) (Schmidt and Guy, 1997; Jensen and Popay, 2007).

*Aploneura lentisci* is endemic to the Mediterranean and Middle East region where it is holocyclic, forming galls on its primary host, *Pistacia lentiscus* (Anacardiaceae), alternating over a 2-year period between *Pistacia* and secondary hosts, principally species of Gramineae (Cottier, 1953; Wool, 2005). This aphid has a wide geographical range on its secondary hosts, on which it exists as permanent, anholocyclic, parthenogenetic populations (Wool and Manheim, 1986). Winged morphs of *A. lentisci* have been trapped in Australia and New Zealand (O’Loughlin, 1962; Lowe, 1968) but have not been observed in the field (A.J. Popay unpublished). Mobile young nymphs can be found on the herbage (Rasmussen et al., 2008a) while mature aphids are largely sedentary living amongst copious amounts of flocculent white wax which likely protects them from soil moisture extremes, microbes and predators. This species is reported to be abundant in grassland in Britain (Purvis and Curry, 1981), occurs throughout New Zealand (A. J. Popay, C. Pennell, D. E. Hume, unpublished observations) and is common in Australian pastures (Salmon et al., 2008; Moate et al., 2012). It has been reported to cause severe damage to young wheat plants (Mustafa and Akkawi, 1987) although Cottier (1953) considered it to be of no economic importance on Gramineae in New Zealand.

We have found no published information on the population dynamics of *A. lentisci* on the roots of its secondary hosts and no direct evidence of its effects on grass growth over an extended period of time. Here we report on populations of *A. lentisci* sampled on individual plants on several occasions in two pot trials. Plant growth has also been measured to test the hypothesis that *A. lentisci* has detrimental effects on host plant growth related to different effects of fungal endophyte strain on population size. One pot trial also investigated the effect of container size on populations after observations indicated aphid colonies were common on the roots growing at the interface between the potting media and the container. From this it was postulated that a greater accumulation of roots at this interface for plants in smaller containers may influence population size and the effects of endophyte strain. Aphid response to ryegrass with and without endophyte to determine if endophytes had a deterrent and/or a toxic effect was also investigated in a series of Petri dish experiments.

## Materials and Methods

The hypothesis that plant growth would be differentially affected by fungal endophyte strain according to the effects of each strain on populations of *A. lentisci* was tested in two pot trials, namely a plant growth trial (PG) and a root mass trial (RM). In both trials root and herbage growth was quantified along with the root aphid populations on individual plants in successive samplings over 2 years in the PG trial and 10 months in the RM trial. In addition, to more closely examine effects of endophyte infection on aphid behavior and population development, aphids were closely monitored on plants for short periods of time in four Petri dish experiments.

### Plant Preparation and Maintenance

In all trials, *L. perenne* cv. Grasslands Samson without endophyte (Nil) or infected with endophyte strains AR1, AR37 or CT were used. All plants were grown from seed obtained from the Margot Forde Germplasm Centre, AgResearch, Palmerston North, New Zealand. Seed was germinated in the dark on damp filter paper in Petri dishes held at 20°C for 5–7 days. Germinated seed was planted into a 2:1 soil:sand mixture into individual pots (120 mm diameter × 100 mm deep) in the PG trial and into a commercial potting mix in polystyrene trays (300 × 500 × 90 mm) for the RM trial and Petri dish experiments. Plants were maintained in ambient light and temperature conditions under automatic overhead watering in a screenhouse. Nutrients were supplied to plants in two forms. At planting Osmocote^®^ slow release fertilizer (19% nitrogen, 2.6% phosphorous, 10% potassium) was incorporated into the top 50 mm of the planting medium at a rate of approximately 2.0 g per plant. Once established, plants received a nutrient solution comprised of a commercially available nutrient mix, Thrive^TM^, prepared at the recommended rate (approximately 8 g per 4.5 L of tap water) with additional nitrogen (approximately 5 g per 4.5 L) in the form of urea (46% nitrogen). Both pot trials were conducted outdoors under ambient conditions. Irrigation was deliberately kept to the minimum needed to prevent the plants from wilting and dying during prolonged dry weather during the summer-autumn period between December and April. During this period, in 2000/01 and 2001/02, respectively, average monthly rainfall was 84.5 and 93.3 mm; minimum monthly rainfall was 28 and 21 mm; average number of rain days was 10.2 and 13.2; average maximum/minimum air temperature was 22.8/12.4°C and 22.3/12.2°C. As required, plants were watered by hand with a hose held for 4 s over each plant, or using a sprinkler.

The endophyte status of all plants used in the trials was determined by taking a single tiller from each 6- to 9-week-old plant to test for the presence of endophyte using a tissue print immunoblot method (Simpson et al., 2012). A tiller was cut near the base, and the freshly cut surface was blotted onto the surface of nitrocellulose paper. A development process was then used that exposed protein produced by the endophyte to polyclonal antibodies resulting in a color change if the tiller was infected with endophyte.

### Plant Growth Trial

**FIGURE 1:**
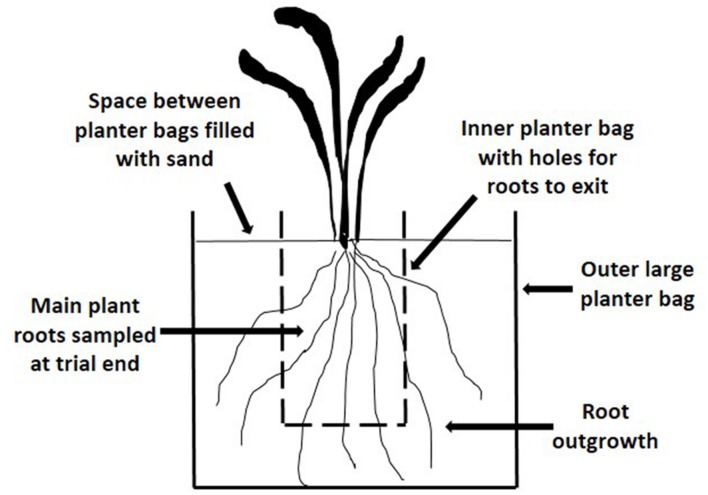
**Diagram of the arrangement for plants in the Plant Growth Trial (PG) to enable root growth and aphid populations to be monitored**.

When plants were 6 months-old, ramets comprising six tillers were planted individually into a soil/sand growing medium (2:1) in polythene planter bags (90 mm × 90 mm × 200 mm). To enable root growth to be measured periodically without disturbing the plant, additional pairs of holes (5 mm diameter, 25 mm apart) were made in each planter bag at 30 mm, 70 mm, and 110 mm from the top of the bag and aligned with existing holes. Twenty replicate plants were arranged randomly on a sand base within a large tub. Initially sand was placed around the planter bags until it was level with the planting medium in the bags. After the first root sampling in August 2000, each plant was isolated from others by placing the small planter bag inside a larger one (160 mm × 160 mm × 370 mm), with the space between each bag filled with sand (**Figure 1**). Root ‘outgrowth’ was determined by severing roots where they exited the holes in the smaller bag into the larger planter bags. Sampling of herbage above 50 mm and root outgrowth was carried out on five occasions; in late winter and early summer 2000, autumn and spring 2001 and in mid-summer and autumn 2002. The plant roots in the small planter bag in which plants were originally planted were also harvested at the final sampling in autumn 2002. Root aphid populations were measured on each occasion.

After a further check of the endophyte status of all plants in late spring 2001, 20 months after the first test, both AR37 plants in one replicate and one in another were found to have lost their endophyte and data for both replicates of this treatment were then excluded from all analyses.

### Root Mass Trial

Although root aphid colonies occur throughout the root system of infested plants, they appeared from casual observations to be more concentrated on roots at the interface between the potting medium and the container. Thus this second trial was designed to investigate the effect of container size and possible interactions with endophyte strain on infestations of root aphid and associated plant growth effects.

To take account of plant genotype/endophyte interactions known to affect root aphids (Popay and Easton, 2006) 6-month-old plants were cloned by taking two ramets of six tillers from each of 15 replicate plants. One of each cloned pair of treatments was planted into a small planter bag (120 mm × 120 mm × 230 mm containing 2484 cm^3^ of 2:1 sand/soil medium) and the other into a large bag (140 mm × 140 mm × 280 mm containing 4900 cm^3^ of 2:1 sand/soil medium). Plants were arranged in two rows with cloned pairs of plants adjacent to each other in separate rows and treatments randomly arranged within each replicate. A square of weed mat was placed underneath each planter bag so that any roots which grew through the base of the bag could also be sampled. Herbage growth was determined by harvesting tillers above 5 mm on seven occasions through the course of the trial. Roots were sampled on three occasions (spring 2002, mid-summer and early winter 2003) by destructive sampling of five replicates of each treatment. Root dry weight and root aphid populations were measured on each sampling occasion.

### Herbage and Root Sampling

For both trials herbage was harvested at 4 cm and root material was captured in a three-stage washing process that was also designed to remove invertebrates from the samples. Roots together with the planting medium were first placed in a bucket and agitated while filling the bucket with water. After a short standing period, the suspension containing the invertebrates was then decanted off while the remaining sand was washed through a 2.5 mm^2^ mesh from which roots were retrieved. Before drying, roots were washed more thoroughly under running water over a 1 mm^2^ mesh to remove any further sand and debris. Root and herbage samples were either frozen and then freeze dried before weighing if they required chemical analysis or oven dried. Herbage samples were oven dried at 60°C for 36–48 h and roots at 80°C for 48–60 h. All samples were weighed immediately after drying.

### Aphid Inoculation and Sampling

Soon after plants were set up in each trial, they were inoculated with root aphids by inserting a small piece of infested root down the side of the planter bag, although it was noted that many plants had become naturally infested prior to this. The number of aphids inoculated was not determined because of the difficulty in doing so and the risk of damaging the aphids. The root aphids were sampled at each plant growth assessment in 2001 and 2002 in the PG trial and at each of the three destructive harvest times in the RM trial. Aphids were extracted by flotation in water and wet sieving. After roots were initially washed in a bucket as described above, the suspension was decanted through three sieves (2.00 mm, 710 μm, and 210 μm). The two larger sieves were rinsed thoroughly but gently before all material that had collected on the 210 μm sieve was washed into a 70 mL specimen container. Samples were stored at 4°C until counting.

For counting, samples were transferred to a beaker and diluted if necessary to give an amount between 30 and 60 mL. The total amount depended on the size of the original sample and the number of aphids present. The sample was stirred thoroughly to distribute the aphids in the sample before a 10 mL subsample was removed to a Petri dish base (90 mm diameter) in five 2 mL aliquots, using a pipette. The base of the Petri dish was marked with a grid (approximately 1 mm^2^) to facilitate counting of the aphids in the dish. Counting was carried out under a stereo microscope at 16× magnification.

### Petri Dish Experiments

Four experiments (A–D) were conducted on plants in Petri dishes to enable regular observations of root aphid behavior and population dynamics on perennial ryegrass with and without endophyte. The effect of plant genotype was also investigated by using cloned plants in Experiments A and B which were tested for their effects on root aphid at different times, and then using cloned plants again in Trial C which were tested concurrently.

For each trial, the base of a 90 mm diameter Petri dish was firmly packed with a 60 mL volume of perlite mixed with 25 mL of tap water and approximately 2.0 g of Osmocote^®^ slow release fertilizer. Plants or tillers from plants were placed in the Petri dishes so that the base of the tiller was level with aligned holes (approx. 10 mm wide) cut in the side of the base and lid of each dish. Roots were splayed out on the surface of the perlite before the lid was put in place and sealed with a 20 mm wide piece of parafilm. Replicate groups of Petri dishes were placed upright in random order and fastened together with a rubber band. A piece of black polythene with a slit in the center where the tillers emerged was placed over each group of Petri dishes to exclude light from the roots and fastened in place with another rubber band. Each replicate was then partially buried in potting mix in a polystyrene planter box and kept outside under ambient light and temperature conditions.

After a period to allow plants to establish, mature and immature root aphids taken from potted plants were transferred with a fine paint brush on or close to roots of the plants in the Petri dishes. Maturity was arbitrarily based on size (immature < 1 mm > mature). Aphids were later checked and replaced if damaged in any way before lids and parafilm were replaced.

To count and observe root aphid in each trial, lids were removed from the Petri dishes and the surface of the perlite and roots were inspected under a stereo microscope (16× magnification). The number of live and dead aphids was recorded and dead aphids were removed. These observations probably underestimated the aphid populations as counting was done with minimal disturbance of the roots and perlite. Location of the aphids on or off roots (on perlite and not in contact with roots) was noted at each inspection in all trials, and their preference for new (i.e., roots grown since planting) or old roots was determined at all assessments in Trial B. In Trial A, 5–10 mL of water was added to each Petri dish at every second inspection, which kept the perlite damp. In subsequent trials water was added only as necessary to maintain the perlite in a moist condition. At the completion of each trial root aphid numbers in each Petri dish were counted. The endophyte status of at least one tiller from each plant was confirmed by staining and microscopic examination.

Trial A: A single healthy tiller was removed from each of five 1-year-old plants of each treatment and planted into separate Petri dishes to give five replicates of each endophyte treatment. One-week after planting, 10 mature and five immature aphids were released onto each plant. The trial was terminated after 25 days.
Trial B: This was planted at the same time as Trial A using clones of the same plants with five replicates of each endophyte treatment. Plants were inoculated with 10 mature and five immature aphids 4 weeks after planting. Petri dishes were inspected regularly for 25 days.
Trial C: For each endophyte treatment, five cloned pairs of plants were tested by taking two ramets of two tillers, matched for root size, from five individual 1-year-old plants and planting them separately into Petri dishes. Four weeks after planting five mature and five immature root aphids were released into each Petri dish. The experiment was assessed for 21 days.
Trial D: This trial tested the effects of the different endophyte treatments in 10-week-old ryegrass plants. They were tested for endophyte before 20 plants of each endophyte treatment were planted into Petri dishes. Four weeks after planting, the 10 healthiest plants of each treatment were inoculated with 12 root aphids, of which at least five were mature and five immature. The Petri dishes were checked regularly for 21 days and then left without checking for a further month during which time they were watered individually as necessary.

### Statistical Analysis

Root aphid numbers/plant and number/g of root (aphid loading) for each of the pot trials were log transformed to stabilise the variance. All log transformations used a constant that was based on the minimum number of aphids possible for each data set based on the dilution of samples prior to counting. For example if the original sample of 20 mL was diluted to 40 mL for counting, one aphid counted in the diluted sample was equivalent to two in the original sample; hence the log transformation was L(*n* + 2). Data were analyzed using a general analysis of variance in Genstat Releases 6.1–17, testing for main effects of endophyte in the PG trial, and plant container size and harvest date in the RM trial. Block strata for the analysis of the PG trial was based on the randomized block design for each replicate of endophyte treatments. Similarly, in the RM Trial, the analyses were structured to take into account the randomized block design of the trial and the cloned plants within each replicate. Cumulative herbage and root dry weight data were analyzed in a similar way but did not require log transformation. In the RM trial, the cumulative herbage growth prior to each root sampling was analyzed separately; i.e., for the first root sampling, three herbage cuts had been taken on all 15 replicates; in the second, five herbage cuts on 10 replicates; at the third root sampling, there were seven herbage cuts on five replicates. Means were separated using Fisher’s protected least significant difference test.

In the Petri dish experiments, an analysis of variance was also carried out on log transformed aphid numbers structured to also investigate the effect of time, and blocked by replicate. Pearson’s correlation analysis in Excel investigated the effect of plant genotype.

## Results

### Plant Growth Trial

**FIGURE 2:**
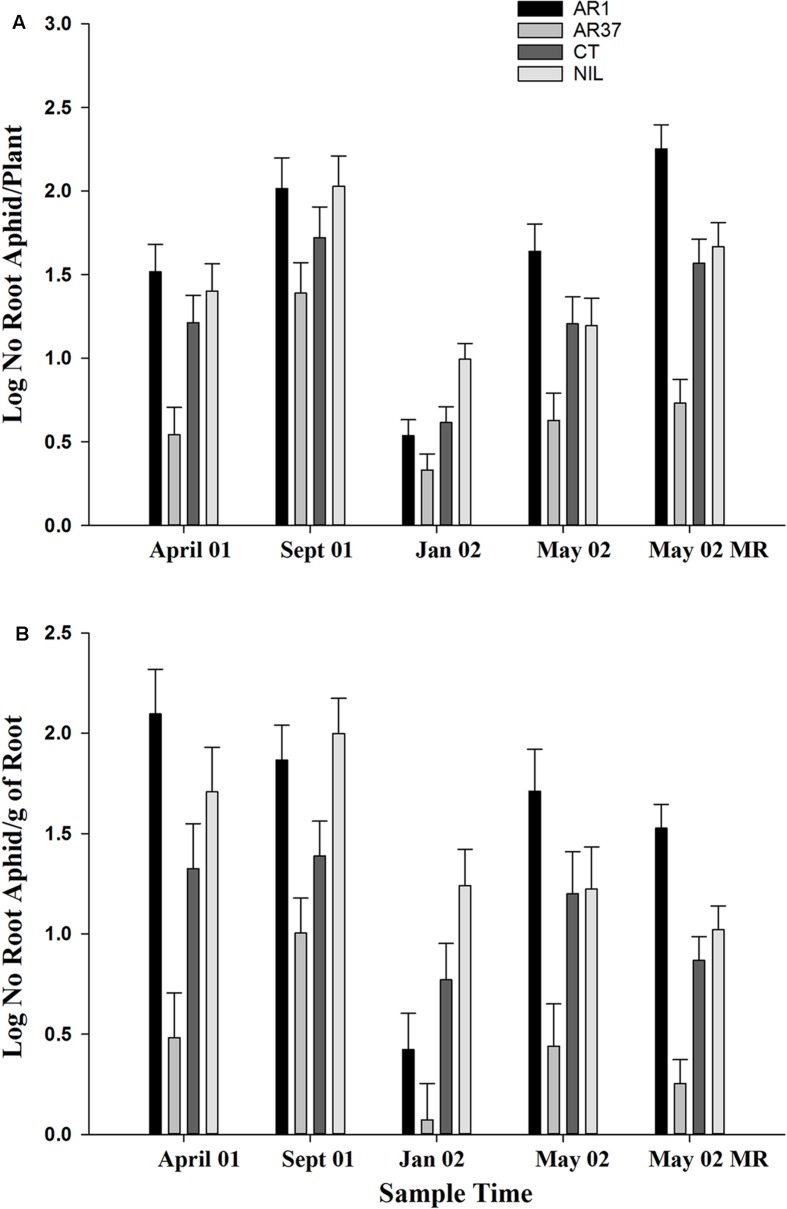
**Effect of different strains of fungal endophytes in perennial ryegrass on *Aploneura lentisci* in the PG trial **(A)** Number of root aphids per plant **(B)** Aphid loading.** Sample times April 01 to May 02 aphids were taken from root outgrowth; sample May 02 was from the main plant roots: Error bars = SED

The most consistent and statistically significant result in this trial was the strong suppression of *A. lentisci* population growth on ryegrass plants infected with AR37. This is shown for both aphids per plant and aphid loadings (number/g of root; **Figure 2**). AR37 had significantly lower populations than the other three treatments (*P* < 0.001) for both aphid numbers per plant and aphid loadings on root outgrowth in April 01 and May 02, and for the main plant roots also sampled in May 02. In September 01, AR37 and CT had fewer aphids per plant (*P* = 0.007) and lower aphid loadings (*P* < 0.001) than AR1 and Nil; in January 02, aphid populations on AR1 were similar to AR37 and both were lower than on Nil, with AR37 populations also lower than CT. The AR1 strain had consistently high infestations of root aphid, except in January 02, with significantly higher loadings than on Nil on three of five sampling occasions. By comparison with Nil treatments, root aphids tended to be less numerous on CT-infected plants but for most samplings this difference was not significant.

Aphid plant populations were highest in September (**Figure 2A**) when actual mean numbers/plant were 515, 55, 269 and 254 for AR1, AR37, CT and Nil, respectively. The average aphid numbers across all samples in the trial including the final plant assessment was 347, 15, 105, and 148/plant. The highest aphid loading occurred on root outgrowth of AR1 plants in April 2001 (**Figure 2B**).

**FIGURE 3:**
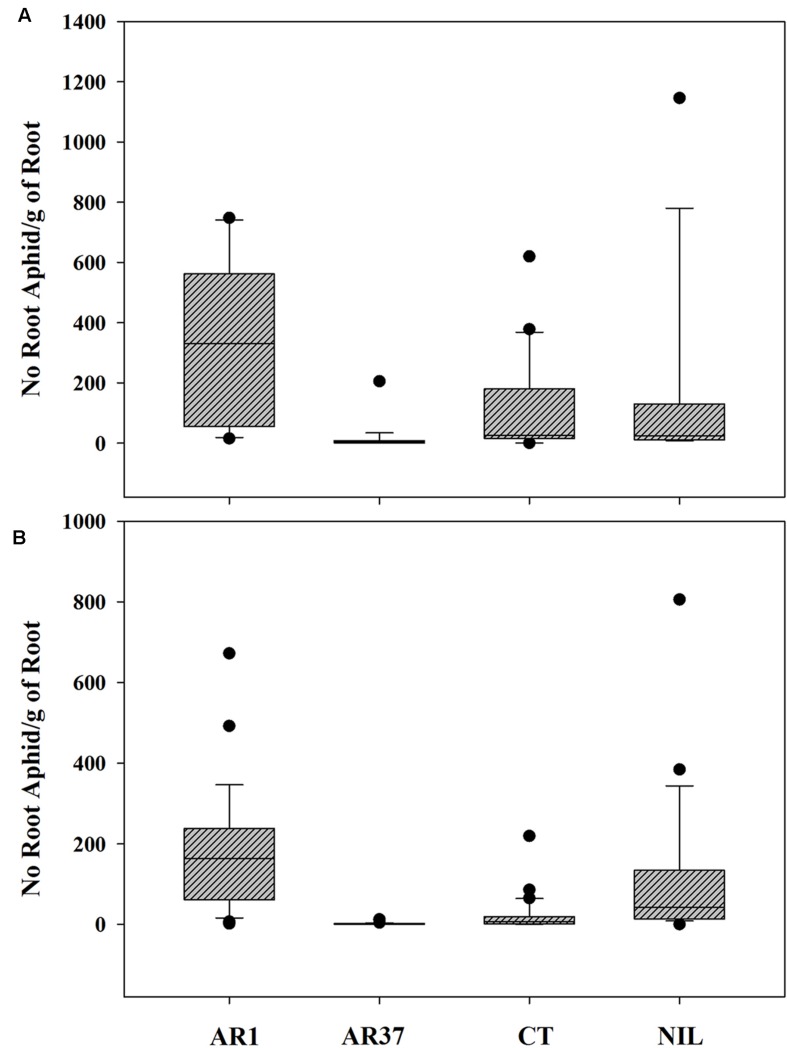
**Variability in root aphid loading per plant among individual ryegrass plants without endophyte (Nil) or infected with AR1, AR37 or CT in the **(A)** September 01 sampling of the PG and **(B)** the three samplings in the Root Mass Trial**.

Root aphid populations varied widely among individual plants infected with AR1 and Nil, varied less on CT but showed little variation on plants infected with AR37. On AR1, aphid numbers ranged from 0 to 1116 on the root outgrowth of different plants at the first autumn sampling, compared with 0–750 for Nil plants, 0–573 for CT and 0–12 for AR37. The same level of variability was also seen in aphid loadings (**Figure 3A**)

Cumulative herbage dry weight from six consecutive harvests during the trial for AR37-infected ryegrass exceeded that of all the other treatments (*P* < 0.001; **Table 1**). In contrast to this, cumulative root growth did not differ (*P* > 0.05) between endophyte strains, although AR37 recorded the highest root dry weight. Likewise, the dry weight of main plant roots in the small planter bag at the final harvest was not significantly affected by endophyte treatment (*P* > 0.05).

**Table 1:** Dry weights (g/plant) of herbage and roots from *Lolium perenne* without endophyte (Nil) or infected with three different endophyte strains from (A) Plant Growth Trial (PG) and (B) Root Biomass Trial.

	AR1	AR37	CT	Nil	SED	df	LSD (5%)
**A. Plant growth trial**							
Herbage	24.1	31.7	25.0	25.5	1.84	44	3.71
Root growth	5.2	7.6	6.8	6.3	0.90	44	NS
Main root	10.5	9.1	8.8	9.2	1.14	123	NS
**B. Root mass trial**							
Herb Sept 02^1^	6.6	9.0	9.9	7.4	0.481	36	0.98
Herb Jan 03^2^	13.4	16.4	18.1	13.1	0.966	24	2.00
Herb Jun 03^3^	20.0	23.7	26.0	19.2	2.289	12	3.43
Root Sept-02	1.6	3.8	4.3	2.5			
Root Jan-03	3.3	4.6	6.9	4.9	1.21	36	2.41
Root Jun-03	3.3	8.5	6.7	6.6			
Root Mean	2.6	5.5	5.8	4.4	0.68	36	1.39


*A: Cumulative herbage and root growth from six samples and dry weight of main roots from plants at final harvest; B: Root biomass taken at three destructive harvests and cumulative herbage growth from successive samples taken up to the time of each harvest. Cumulative herbage dry weights from ^1^three samples taken from 15 replicates, ^2^five samples taken from 10 replicates; ^3^seven samples taken from five replicates.*

### Root Mass Trial

Over all assessments, AR37 had fewer aphids/plant and lower aphid loadings than all other endophyte treatments (*P* < 0.05) while there were more aphids and greater aphid loadings on AR1 and Nil than on CT (*P* < 0.05; **Table 2**). Aphid populations per plant for AR1 and Nil did not differ significantly whereas aphid loading across all harvests was greater on AR1 than on Nil (*P* < 0.05).

**Table 2:** Root Mass Trial (RM) Effect of *L. perenne* without endophyte (Nil) or infected with AR1, AR37, and CT on overall mean aphid populations and aphid loading/plant (pooled for both planter bag sizes) and for each harvest date.

	Mean		September 02		January 03		June 03	
				
	Log^1^	Actual^2^	Log	Actual	Log	Actual	Log	Actual
**No/plant**								
AR1	2.338	406	2.261	315	2.7	605	2.054	296
AR37	0.872	6	1.075	11	0.788	3	0.753	3
CT	1.596	137	1.25	19	1.899	258	1.637	133
NIL	2.215	382	2.276	555	1.973	172	2.397	419
SED	0.1789		0.3271		0.3271		0.327	
df	36		46		46		46	
LSD (5%)	0.3628		0.2680		0.2680		0.2680	
**No./g/of root**								
AR1	2.037	173	2.125	234	2.258	198	1.727	88
AR37	0.715	2	0.820	4	0.680	1	0.646	1
CT	1.108	21	0.902	5	1.322	44	1.101	16
NIL	1.681	102	1.935	214	1.422	35	1.685	57
SED	0.1297		0.2377		0.2377		0.237	
df	36		46		46		46	
LSD (5%)	0.2630		0.4784		0.4784		0.4784	


*^1^Analysis carried out on log (*n* + 4) transformed data.*

*^2^Arithmetic means.*

Container size had no significant effect on root aphid populations either for individual endophyte treatments or overall (*P* > 0.05). In a significant interaction between endophyte and container size, however, aphid loadings for AR1 plants were greater in small containers than in the large ones [Log No.aphids/g of root: Small 2.220, Large 1.853; LSD (5%) 0.3060, df 46 *P* < 0.05]. For the other endophyte strains, aphid loadings were very similar (respectively, for small and large containers Log No./g of root: AR37 0.690, 0.740; CT 1.201, 1.016; Nil 1.649, 1.712).

There was a significant effect of harvest time on root aphid populations but no significant interaction between endophyte and harvest date. There were more aphids on AR1 and CT plants sampled in January 2003 than at other harvests, although aphid loadings showed less seasonal variation for these two treatments. In Nil plants the highest aphid populations and loadings occurred in September 2002. Over all treatments aphid loadings were lowest in June.

The extreme variability in root aphid numbers per plant which characterized the PG trial was also evident in this trial for AR1 and Nil plants (**Figure 3B**). Individual plants within each treatment had been cloned between the large and small containers enabling the role of host plant genotype to be explored. An analysis of log transformed aphid loading for each clonal pair of plants within each treatment showed significant correlations between the cloned pairs of CT and AR1-infected plants (Pearson’s correlation coefficient 0.77 and 0.76, respectively; *P* ≤ 0.002) whereas neither parameter was correlated in Nil plants (Coefficient 0.47; *P* > 0.05).

For all three destructive harvests of this trial the cumulative total herbage dry matter removed was significantly higher from CT and AR37-infected plants than from Nil and AR1 (*P* < 0.001; **Table 1**). At all three time points, cumulative herbage dry weight from plants growing in the larger planter bags was significantly greater than that in the small bags with no significant interactions between this and endophyte treatment (data not presented). Endophyte effects on mean root dry weight was similar to the effects on herbage, with the overall mean root weight from the three harvests greatest in CT and AR37 treatments and significantly (*P* < 0.001) more than for Nil and AR1 with the latter two also significantly different (**Table 1**). Root weights were significantly greater for plants grown in larger planter bags than the small ones in all endophyte treatments.

### Petri Dish Experiments

**FIGURE 4:**
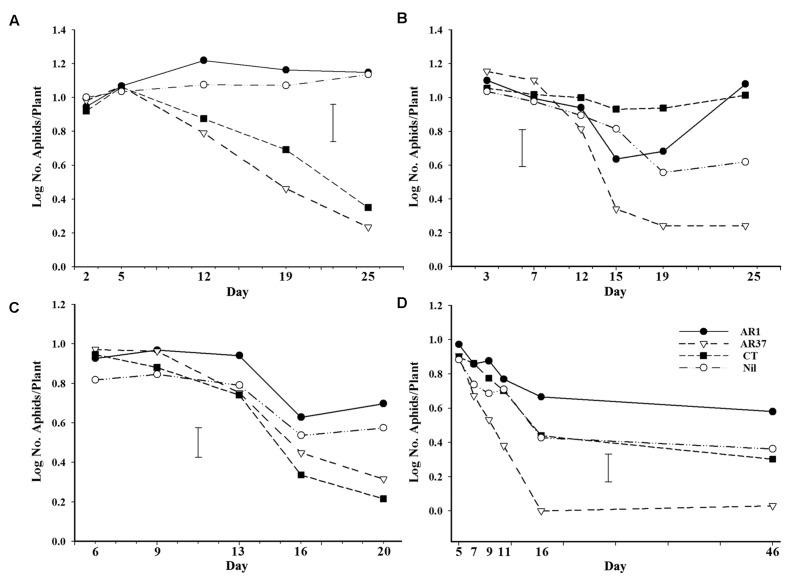
**Numbers of root aphid/plant on ryegrass plants without endophyte or infected with AR1, AR37, or CT at different times after inoculation with aphids in Petri dish experiments **(A–D)**.** Error bars = SED.

There were overall effects of endophyte in each trial, with AR37 having significantly fewer aphids than at least one other treatment in each trial (*P* < 0.02) and, likewise, CT also having low numbers in Trials A and C. On plants infected with AR37, root aphid survival declined to very low levels in all four trials, after an initial phase in which numbers were similar in all treatments (**Figure 4**). In Trials A and C root aphid numbers on CT plants followed a similar pattern of decline to those on AR37 whereas in Trials B and D aphid performance on CT was similar to that on AR1 and Nil treatments. Differences between AR37 and other treatments did not become significant until Day 19 in Trial A, Day 15 in Trial B, Day 20 in Trial C, and Day 7 in Trial D. In Trial A, it was also Day 19 before CT had significantly reduced numbers compared with AR1 whereas in Trial C it was Day 16, slightly earlier than AR37.

The role of plant genotype was considered by comparing aphid performance on cloned plants in Trials A and B and again in Trial C. For AR1, final numbers/plant were highly correlated between individual cloned plants in Trials A and B and again between the cloned plants in Trial C (**Table 3**). For CT-infected plants the strongly contrasting differences in aphid performance between Trials A and B showed no evidence of a plant genotype effect while in Trial C plant genotype effects could not be tested for when aphid numbers fell to low levels on all five plants. Aphid numbers on Nil plants were not correlated between either Trials A and B (-0.44) or in Trial C (-0.22).

**Table 3:** Petri Dish Experiments Effect of plant genotype on root aphid: final number of aphids/plant for cloned pairs of ryegrass plants infected with AR1 tested at two different times in Experiments A and B and at the same time in Experiment C.

Rep	Trials A and B – Cloned Pairs		Trial C – Cloned Pairs	
		
	A	B	A	B
1	2	0	0	1
2	59	35	38	23
3	46	15	7	6
4	27	10	12	0
5	1	2	0	6
Correlation^1^	0.96		0.87	


*^1^Pearson’s correlation coefficient.*

A large majority of root aphids were located on roots regardless of treatment or aphid maturity, providing no evidence of deterrent effects of AR37 or CT (**Table 4**). There was no effect of assessment time on this aspect (data not presented). In Trial B, both nymphs and mature aphids also displayed a marked preference for new roots in all treatments and at all assessments.

**Table 4:** Petri Dish Experiments Percentage of immature and mature root aphids located on roots (rather than away from roots) in Experiments B and C and on new roots (rather than old roots) in Experiment B.

	Trial	Endo	Immature	Mature	Mean	*N^1^*
% Aphids on roots	B	AR1	86.7	89.0	87.6	315
		AR37	77.6	76.0	77.1	153
		CT	85.7	82.5	84.6	331
		Nil	88.7	84.7	87.1	248
	C	AR1	93.6	91.3	92.8	470
		AR37	89.7	95.5	91.9	357
		CT	84.7	91.7	87.9	256
		Nil	92.7	90.9	91.9	385
% Aphids on new roots	B	AR1	98.0	85.7	93.2	161
		AR37	83.5	80.8	82.5	149
		CT	89.0	84.2	86.9	130
		Nil	89.3	68.3	86.5	144


*N^1^ - Number of aphids observed*

On Day 7 in Trial B, two aphids on separate AR37 plants were trembling quite violently and continued to do so over the ensuing 24 h period. Both had died within 36 h of the time they were first observed. Over that period both aphids remained stationary, one with its stylet inserted into the root throughout. Following this, aphids in other experiments were closely observed and others were also found to be trembling and their movements uncoordinated but only in AR37 treatments. No aphids were subsequently found with tremors as severe as those first observed. Trembling aphids were recorded at Day 5 in Trial D but not until Day 13 in Trial C.

## Discussion

Interactions between insect herbivores and their host plants at any one time depend on host quality, defined by Leather (1994) as “those plant attributes, chemical or physical, that contribute either negatively or positively to the fitness of the insect population or individual insect that feeds upon the plant’s tissues.” Insect performance is therefore governed by a balance between those chemical factors that positively influence its fitness and those that have a negative effect while other elements of host quality include resource availability. *Epichloë* endophyte infection of grasses changes the host quality in terms of its chemistry for those insects that utilize the infected plant as a food source. Differences in chemistry, however, may go beyond the presence or absence of certain alkaloids with more fundamental changes in the plant hosting the endophyte (Rasmussen et al., 2008a,b). The response of any one insect species can vary from negative, where the presence of alkaloids impair the performance of the insect, to neutral, where the insect is not affected to positive, where insect fitness appears to be better on infected plants than on uninfected (Saikkonen et al., 1999; Bultman and Bell, 2003). Effects may be endophyte-strain specific and be transitory rather than stable.

The effects of host quality on insect performance are exemplified in the results of the trials with *A. lentisci*. Populations of this aphid exhibited a marked response to host ryegrass plants ranging from negative to positive that were driven not only by the presence or absence of *Epichloë* infection but also by the strain of endophyte. At the negative end of the scale, ryegrass infected with AR37 was highly resistant to *A. lentisci*. This effect was stable, showing only minor seasonal variation with some increases in populations in spring but little variation in the level of resistance among individual plants. At the other end of the spectrum, ryegrass infected with AR1 was often more susceptible to root aphid than endophyte-free plants. Aphid populations were highly variable on AR1 both on individual plants and over time. In addition, ryegrass infected with other endophytes chemically similar to AR1 have shown similar levels of vulnerability to this aphid (Popay and Gerard, 2007). For Nil plants there was considerable inter-plant and temporal variation in the number of root aphids/plant, and overall aphid performance on this treatment could be considered to range from neutral to positive. Aphids tended to be less numerous on CT than on Nil plants but not always significantly so. Thus aphid performance on ryegrass with CT endophyte was mostly neutral with what appears to be transient negative effects. Inter-plant and temporal variations in number of aphids on CT were much less than on Nil and AR1.

The amount of roots may provide one explanation for differences between treatments but aphid loadings generally reflected the numbers/plant and did not change relative differences between endophyte treatments suggesting that this was not a limiting factor. As a measure of resource availability, however, root weight may not be sufficient because it takes no account of differences in root morphology and age which may be equally, if not more important, for aphid performance. This was evident from the Petri dish experiments in which aphids exhibited a strong preference for new roots suggesting that the availability of new roots, rather than the total root weight *per se*, is more important for population development. In this regard, the design of the pot trials in allowing repeat sampling of new root growth was useful. The strong preference to inhabit young roots also suggests that actively growing plants are likely to stimulate population growth. Growth affects the quantity and quality of phloem, both of which are factors that contribute to aphid performance (Whitham, 1978). The preference root aphid showed for new roots may be explained by changes in chemistry as roots age but equally may be due to physical factors such as increasing lignification that may make it difficult for the aphid to probe older roots. Respiration rates are higher and uptake of nutrients and water more efficient in new than in old roots (Eissenstat and Yanai, 1997; Bouma et al., 2001) but there is little other information on physiological changes in maturing roots that may explain aphid preference.

If habitat was not limiting aphid populations then plant chemistry is the most likely basis for the differences observed among endophyte treatments. The effects of AR37 on *A. lentisci* were most likely attributable to the production of a metabolite by the fungus that was toxic to the aphid. The tremors induced when the aphid feeds on plants infected with AR37 indicated that the compound was a neurotoxin. In all the Petri dish trials there was an initial phase lasting up to 2 weeks after aphids were released onto the plants in which the aphid behavior, feeding and reproduction appeared normal. Such a delayed effect suggests that the toxin was either a slow-acting constitutive compound or one that is inducible. The proportion of aphids recorded on roots provided no evidence of a deterrent response to AR37.

The effect of the CT strains may also be due to the presence of a secondary metabolite. In Experiments A and C in Petri dishes, the rapid decline in aphid numbers on CT was symptomatic of the presence of a toxin but there was no indication of this in Experiments B and D. Plants in Experiment B were clones of those in A, ruling out plant genotype as a factor in the different population responses. Plants in B and C had been grown in the Petri dishes for a similar length of time prior to inoculation with aphids and were kept under similar ambient conditions. Experiment C was conducted a month after Experiment B in spring when temperatures were warmer (mean maximum/minimum temperatures were: B 15.2/5.3°C; C 17.0/7.0°C) but there was no indication in the pot trials that aphid performance on CT varied with seasons or temperature. The alkaloids produced by CT endophyte with known anti-insect activity are lolitrem B, ergovaline and peramine (Popay and Bonos, 2005). Peramine was ruled out as affecting aphids since it is the only one of the three compounds that is also produced by AR1. In an experiment by Popay and Gerard (2007) that compared endophytes with different alkaloid profiles, ergovaline was implicated in low root aphid populations although the reason for the transient effects observed is unknown. Ergovaline concentrations in plants vary seasonally and with environmental conditions (Ball et al., 1995a; Lane et al., 1997) and are also linked to plant genotype (Easton et al., 2002). Despite being a lipophilic compound this alkaloid does occur in roots in concentrations that can be as high as those in the pseudostem in ‘Grasslands Samson’ infected with CT (A. J. Popay unpublished).

A strong host plant genotype influence on aphid fitness on CT and AR1-infected plants was previously reported for the PG trial by Popay and Easton (2006). A similar analysis of aphid populations on the cloned plants in small and large planter bags in the RM trial also provided evidence of a plant genotype effect but again only for those plants infected with CT and AR1. The weakness of the link between plant genotype and aphid performance in Nil indicates that a host plant genotype/endophyte interaction may be moderating aphid performance more than plant genotype itself. A similar high degree of variability associated with inter-plant genotypic differences has been found in the amount of damage inflicted on AR1-infected plants by black beetle adults (Easton et al., 2000). Alkaloid production is linked to endophyte concentration in the plant and is markedly influenced by host plant/endophyte interactions (Ball et al., 1995a,b; Easton et al., 2002). Other aspects of plant growth and mineral uptake have also been shown to vary according to interactive effects of endophyte and host plant genotype (Malinowski and Belesky, 1999; Malinowski et al., 2000; Cheplick and Cho, 2003).

Composition and concentration of amino acids and concentration of sucrose in the phloem are important determinants of aphid performance (Douglas, 1993; Karley et al., 2002) and levels of soluble nitrogen are often causally linked to inter- and intra-plant differences in aphid fitness, site preferences, host alternating behavior and seasonality (Leather, 1994). Endophyte infection of *L. perenne* can modify the metabolic profiles of their hosts, interacting with nitrogen supply and host plant genotype, in ways that influence herbivore response (Rasmussen et al., 2008a,b). Differences in some of these factors may account for not only the apparent differences in aphid performance between AR1 and Nil, but also the extreme variability between individual plants.

Unlike many foliar-feeding aphid species, there was no discernible pattern in aphid numbers over time or season. In the PG trial, numbers were highest in autumn 2001 but then fell to very low levels in summer 2002. Seasonality was not the cause since aphid populations were generally high in the RM trial in summer 2003. Anderson (1987) noted that root herbivores are often chronic pests and this would appear to be true for *A. lentisci*. Observations also suggest that, like many root herbivores, *A. lentisci* are highly aggregated in their distribution, forming sometimes large colonies on roots where they cocoon themselves in wax secretions. They show no preference for a particular depth in the soil profile but exploit large pore spaces in the soil structure where there is often a proliferation of new roots; hence their apparent prevalence at the interface between the growing medium and container. At times in the field and in potted plants, aphids have been observed feeding at the soil surface, clustered around the base of tillers. A similar behavior in another root aphid *Pemphigus bursarius* on lettuce plants has been associated with the production of sexupariae that develop into winged morphs in readiness for flight to their primary host (Dunn, 1959). Winged *A. lentisci* were not observed in the course of this study but have been trapped in both New Zealand and Australia (O’Loughlin, 1962; Lowe, 1968). Early instar nymphs are highly mobile in the soil and have also been sampled from the foliage of ryegrass (Rasmussen et al., 2008b). Thus dispersal mechanisms are likely to involve movement of these nymphs along the surface but, given their very small size, may also include dispersal by wind.

There is considerable information in the literature on the detrimental effects of foliar aphids on plant growth and fitness (e.g., Giménez et al., 1997; Riedell et al., 2007) but much less information on root aphids. Hutchison and Campbell (1994) demonstrated a severe effect of the sugar beet root aphid, *Pemphigus betae* on yield and sugar content including a 54% reduction in total recoverable sugar/ha. Similarly the lettuce root aphid, *P. bursarius*, and the cabbage root aphid *Pemphigus populitransversus* cause significant economic yield loss (Royer and Edelson, 1991; Liu et al., 2011). Here, using comparisons of *L. perenne* growth between the resistant AR37 and the other treatments in the PG trial, *A. lentisci* reduced overall foliar growth by between 20 and 23% but did not reduce cumulative root growth. In addition to this, however, survival of AR1 and Nil plants was reduced by 35% whereas there was no mortality of CT and AR37 plants during the trial (data not presented). In autumn and spring 2002, tillers of AR1 and Nil plants were also infested with a mealybug (data not presented) in the PG trial but given that cumulative foliar growth on CT was similar to that for Nil and AR1, root aphid can be considered to be the primary factor affecting this. In the RM trial, herbage growth of AR1 and Nil was 16–27% less than AR37 over the different harvests while average root mass of AR37 over the three harvests was over 50% greater than for AR1 and 22% greater than Nil. In contrast to the PG trial, growth of CT plants has matched or exceeded that of AR37. This difference between the two trials reflects a difference in root aphid loadings which were considerably lower on the CT treatment in the RM than in the PG trial (mean 21 cf. 142/g of root). Effects of infestations of *A. lentisci* have also been demonstrated in the field (Hume et al., 2007) where very high populations of aphids can occur causing a chronic loss of vigor and large yield loss of ryegrass. The presence of this aphid year round on plants will be a constant drain on the plant’s resources, resulting not only in reduced plant performance but also poor survival. Thus the results reported here have supported the hypothesis that differences in plant growth of *L. perenne* without endophyte or infected with different strains of endophyte are associated with their effects on populations of *A. lentisci*.

## Author Contributions

AP designed and carried out all the experimental work reported here; NC provided statistical expertise.

## Conflict of Interest Statement

AP is a patent holder for AR37 and receives research funding from the IP owner Grasslanz Technology Ltd, and licensee, PGG Wrightson Seeds. AP also receives a share of royalties from the sale of AR endophytes. The other author declares that the research was conducted in the absence of any commercial or financial relationships that could be construed as a potential conflict of interest.
